# Properties of an acrylic resin after immersion in antiseptic soaps: Low-cost, easy-access procedure for the prevention of denture stomatitis

**DOI:** 10.1371/journal.pone.0203187

**Published:** 2018-08-30

**Authors:** Jacqueline de Oliveira Zoccolotti, Camilla Olga Tasso, Maria Isabel Amaya Arbeláez, Isadora Ferreira Malavolta, Eduarda Carolina da Silva Pereira, Caroline Stefanie Gomes Esteves, Janaina Habib Jorge

**Affiliations:** Department of Dental Materials and Prosthodontics, São Paulo State University (UNESP), Dental School, Araraquara, São Paulo, Brazil; Virginia Commonwealth University, UNITED STATES

## Abstract

Denture stomatitis triggered by *Candida* species requires better preventive measures. This study evaluated the physical and biological properties of a denture base acrylic resin after immersion in antiseptic soaps. Acrylic resin specimens were prepared and stored in distinct solutions for 0, 7, 14, 21, and 28 days. The solutions were as follows: DW: distilled water at 37°C (control group); DS: cycles of daily immersion in Dettol soap for 8 hours at room temperature, followed by immersion in distilled water for 16 hours at 37°C; PS: cycles of daily immersion in Protex soap, as described for the previous group; LS: cycles of daily immersion in Lifebuoy soap, as described for the DS group. The parameters evaluated at each time point were the following: biofilm formation capacity by *Candida albicans* and reduction of preformed fungal biofilms, cytotoxicity, surface roughness, hardness, and color change. For the fungal adhesion phase, the type of soap had a statistically significant effect (p = 0.0292), but after 24 hours, no differences were found between solutions or between storage times. Regarding the efficacy of biofilm reduction, there was a significant difference when the groups were compared to each other (p = 0.014). Dettol and Lifebuoy eliminated the preformed biofilm on the specimens. Moreover, all the soaps were classified as non-cytotoxic (on HaCaT cell line) because there was no difference in cell viability between the different groups, except after 21 days, when a decrease in cell viability occurred, regardless of the type of soap. Regarding the roughness, there was no statistically significant difference (p > 0.05) between the groups. Lifebuoy decreased resin hardness regardless of storage time (p = 0.003). After 21 and 28 days of storage, there was an increase in hardness value, regardless of the type of soap. The specimens’ color, according to the National Bureau of Standards values, ranged from 0.27 to 0.58 (i.e., imperceptible or mild color changes). In general, the disinfectant soaps were not able to prevent biofilm formation, but all the soaps were effective in reducing the preformed biofilm. In addition, all soaps were non-cytotoxic and did not change surface roughness, hardness (except Lifebuoy), and color (except Lifebuoy). Therefore, immersion in two antiseptic soaps (Protex and Dettol) may be a cheap and easy procedure for preventing denture stomatitis.

## Introduction

Microbial biofilm, ill-fitting dentures, continuous prostheses use, and local and systemic conditions such as xerostomia and diabetes are predominant factors in the pathology of denture stomatitis [1–6], which is an inflammatory condition of extremely variable etiology, considered multifactorial, found most frequently in patients wearing dentures. The adhesion of microorganisms, mainly the *Candida* species, to the inner surface of the denture bases is considered a main factor in the appearance of this alteration. Hygiene care is an important aspect of the oral health maintenance of denture wearers [7,8] and can be performed mechanically, chemically, or by a combination of both [9].

The most common mechanical procedure for removal of the biofilm on the surfaces of the prostheses used by the patients is brushing with soap or dentifrice [7]. The effective removal of the biofilm by the brushing is considered doubtful because the base surfaces of the prostheses are irregular and porous. In addition, although brushing is the most widely used method, patients with compromised motor coordination have difficulty performing this procedure adequately [9,10].

Thus, chemical cleaning, such as immersion of dentures in disinfectant solutions, should also be performed [11]. Disinfection strategies using chemical solutions have often been recommended because of their effectiveness in inhibiting or eliminating different microorganisms [12]. Several chemical cleaning agents are used for the disinfection or reduction of the biofilm of prostheses, such as sodium hypochlorite, chlorhexidine digluconate, and alcohol [13,14]. Sodium hypochlorite may be useful for the disinfection of prostheses because it inactivates bacterial biofilm and inhibits calculus formation [15,16]. Chlorhexidine, in addition to inactivating the biofilm, reduces the adhesion capacity of the microorganisms, which is considered effective in the disinfection of prostheses [17].

However, studies have shown some disadvantages regarding the use of such disinfectants [18–21]. Although glutaraldehyde has a bactericidal action, it should not be used for the immersion of dentures because it may become impregnated in the porosities of the resin, resulting in an irritant effect on the buccal tissues [18]. Sodium hypochlorite can have moderate cytotoxic effects on oral tissues, as well as promoting acrylic base bleaching and corrosion of metallic components of dentures [19]. Other disadvantages associated with disinfection by chemical agents are reduction of flexural strength after immersion in alcohol [20], low antimicrobial effectiveness of iodophors [21], and staining of prostheses by use of solutions based on chlorhexidine [20]. Currently, antiseptic soaps have been widely used to eliminate microorganisms from various surfaces. Efficacy as an antimicrobial agent has been consistently proven. Antiseptic soaps are capable of removing 65–85% of microorganisms on human skin [22,23]. Taking into consideration the disadvantages of the chemical cleaning agents used for the disinfection or reduction of biofilm on the dentures, it was judged necessary to evaluate the physical (roughness, hardness, and color change) and biological properties (biofilm formation and cytotoxicity) of a denture base acrylic resin after immersion in antiseptic soaps, as well as the ability of these solutions to decrease the *Candida albicans* biofilm of these surfaces. In addition, the effect of immersion time was also evaluated. It is also important to emphasize the ease of the proposed method for the denture disinfection, especially for elderly patients with poor manual dexterity. Immersion of prostheses in liquid disinfectant soaps may be protocol in hospitals and nursing homes for the care of the prostheses of institutionalized and hospitalized patients. The null hypothesis of this study was that the immersion in antiseptic soaps would not change the properties of an acrylic resin.

## Materials and method

The brands and composition of the disinfectant soaps used in the present study are described in Table 1.

**Table 1:** Brands and composition of the antiseptic soaps according to the manufacturers.

Brands and Composition		
Protex®	Dettol®	Lifebuoy®
Aqua, Sodium Laureth Sulfate, Cocamidopropiyl Betaine, Glycol Distearate and Sodium Laureth Sulfate and Cocamide MEA and Laureth-10, PPG-2 Hydroxyethyl Cocamide, Decyl Glucoside, Laureth-7, Parfum, Citric Acid, Sodium Benzoate, Polyquatemium-7, Triclocarban, Tetrasodium EDTA, Poloxamer 124, Fucus Vesiculosos Extract, Sodium Chloride, Methylchloroisothiazolinone and Metylisothiazolinone, Hexyl Cinnamal, Limonene, Butylhenyl Methylpropional, Citronellol	Aqua, Sodium Laureth Sulfate, Cocamidopropiyl Betaine, Salicylic Acid, Chloroxylenol, Glycerin, Polyquaternium-7, Tetrasodium EDTA, Methylchloroisothiazolinone & Methylisothiazolinone, Acrylates/PEG-10 Maleate, Styrene Copolymer, Parfum, Sodium Hydroxide, Citric acid, Sodium Chloride	Aqua, Sodium Laureth Sulfate, Cocamidopropiyl Betaine, Parfum, Cocamide MEA, Acrylates Copolymer, Sodium Chloride, PPG-9, Lactic Acid, Glycol Distearate, Glycerin, Helianthus Annuus Seed Oil, Sodium Hydroxide, Tetrasodium EDTA, Styrene/Acrylates Copolymer, Dried Cream, Terpineol, Thymol, Methylchloroisothiazolinone, Methylisothiazolinone, Alpha-Isomethyl ionone, Benzyl Salicylate, Butylphenyl Methylpropional, Citronellol, Coumarin, Geraniol, Hexyl Cinnamal, Limonene, Linalool

### Fabrication of acrylic resin specimens

Disc-shaped samples (14 mm x 1.2 mm) of a denture base acrylic resin (Vipi Wave: Dental Vipi Ltda) were fabricated and polymerized according to the manufacturer’s recommendations. After polymerization, excess resin from the processing was removed using a sterilized trimming bur and the specimens were stored in water for 48 hours (zero time), immersed in distilled water at 37°C to remove excess monomer. After storage for 48 hours, the surface roughness was measured to standardize the specimens and to homogenize the groups. The specimens that had roughness between 2.7 and 3.7 μm were selected for the following tests [24].

### Minimum Inhibitory Concentration (MIC) and Minimum Fungicidal Concentrations MFC

The microorganism used in this study was an American Type Culture Collection strain of *C*. *albicans* (ATCC 90028). The cells were stored in a freezer at -80°C until the assays were performed. For the tests, 25 μL of *C*. *albicans* were seeded in Petri dishes containing the culture medium Sabouraud Dextrose Agar (SDA) with 5% of chloramphenicol, and the plates were incubated at 37°C for 48 hours. Yeast colonies were inoculated into 10 mL of Yeast Nitrogen Base (YNB) and grown overnight aerobically at 37°C for 16 hours. Next, 0.5 mL of the starter culture was transferred to 9.5 mL of fresh YNB. The optical density was evaluated, and the culture was grown aerobically at 37°C for 8 hours. To obtain the standardized suspensions of *C*. *albicans*, each culture was harvested after centrifugation at 4000 rpm for 5 minutes, washed with phosphate-buffered saline (PBS) two times, and re-suspended in Roswell Park Memorial Institute (RPMI) medium to 10^6^ colony-forming unit per milliliter (CFU/mL) by adjusting the optical density of the suspension at 540 nm.

Two-fold serial dilutions of each soap were performed using 12 Eppendorfs tubes with saline solution (0.9% NaCl). After adjusting the concentrations, aliquots of 10 μL of the suspension obtained for *C*. *albicans* were placed in a 96-well plate, and 200 μL of the dilutions of each soap were added into each well, in triplicate. The plate was incubated at 37°C for 24 hours. Microbial growth in each well was visually analyzed to determine MIC. The wells without visual growth were plated to determine MFC and were put into an incubator at 37°C for 24 hours. After this period, the CFUs were counted on the plates. MFC was confirmed in the well where there was no microorganism growth after plating. Each experiment per liquid soap disinfectant was carried out on three separate occasions.

### Experimental groups

After MIC determination, the specimens were divided into groups according to the immersion solution:

DW: The properties were evaluated at 0, 7, 14, 21, and 28 days of immersion in distilled water at 37° C, with distilled water being changed daily (control group);
DS: The properties were evaluated at 0, 7, 14, 21, and 28 days, with daily immersion cycles in Dettol solution (in the MIC obtained);
PS: The properties were evaluated at 0, 7, 14, 21, and 28 days, with daily immersion cycles in Protex solution (in the MIC obtained);
LS: The properties were evaluated at 0, 7, 14, 21, and 28 days, with daily immersion cycles in Lifebuoy solution (in the MIC obtained).

According to Shay et al. [25] and Cakna et al. [26], some disinfectant solutions are more effective when used overnight. Therefore, the protocol used in this study consisted of immersing the samples in solutions for 8 hours per day over 28 days, simulating night disinfection. For the DS, PS, and LS groups, the specimens were incubated for 8 hours at room temperature in each of the corresponding soaps, followed by immersion in distilled water for another 16 hours at 37° C, simulating the night disinfection of the dentures. The disinfectant solution and the distilled water were changed daily.

Due to the high dilution performed to obtain the MICs, the solutions remained transparent (colorless), similar to water. Even so, care was taken to use only white and/or clear neutral soaps available on the market.

### pH measurements

The pH values of the dilutions used in this study were measured using a digital pH meter (Quimis, model Q400AS).

### Biofilm formation capacity

The *C*. *albicans* cultures were performed as described for MICs. For the analysis of the biofilm formation capacity, specimens from each experimental group (n = 6) were individually placed in a 24-well plate containing 750 μL of the aliquot of RPMI medium with *C*. *albicans* and were incubated for 90 minutes at 37°C (adhesion phase) under agitation at 76 rpm. After that time, the liquid cultures were removed from the wells, which were washed twice with 1500 μL of PBS to remove unbound cells. Three specimens from each experimental group were removed from the contaminated wells and transferred to a new plate to evaluate how the tested soaps interfered with the adhesion phase (i.e., prevented adhesion of yeast cells). For other samples, 1500 uL of fresh RPMI medium was added and the plate was incubated for 24 hours for the formation of mature biofilm (the RPMI medium being renewed after 12 hours).

The specimens removed from the contaminated wells and transferred to a new plate received 150 uL of sterile saline. Then, the specimens were scraped with a sterile pipette tip for 1 minute to peel off the formed biofilm. Subsequently, four 10-fold dilutions were made from this final solution. For this, an aliquot of 100 μL of the final solution was pipetted and transferred to an Eppendorf tubes containing 900 μL of sterile saline. This last tube was vigorously vortexed, and a fresh aliquot of 100 μL was removed therefrom and placed in another eppendorf containing 900 μL of saline. This procedure was performed three times for each test specimen; thus, serial dilutions of 10^−1^ to 10^−4^ were obtained. Twenty-five microliters of the 10^−3^ dilution and the 10^−4^ dilution were plated on petri dishes containing the SDA culture medium with 5% of chloramphenicol, and the plates were incubated at 37°C for 48 hours. The same procedure was performed with the specimens that were incubated for 24 hours for biofilm formation. The procedures were performed after the adhesion phase (time 0) and after 24 hours, in triplicate. After incubation, the cell viability (CFU/mL) was determined.

### Alamar Blue® assay

Cell viability of the *C*. *albicans* biofilm was also monitored using the Alamar Blue*®* assay, which is a simple and rapid test in which 10% of the commercially available solution is added to the cell medium and measured either by colorimetry or fluorimetry [27]. However, higher sensitivity is achieved using the fluorescent property. In addition, Alamar Blue is nontoxic to cells, and it is not necessary to eliminate cells to obtain measurements, as occurs with the MTT test [28]. After 24 hours, 20 μL of Alamar Blue diluted in 100 μL culture medium was added to each well. The plate was incubated for an additional 4 hours. The fluorescence of the samples was measured using Fluoroskan Ascent (Lab Systems) at 560 nm (A560) and 590 nm (A590). All experiments described above were performed on three different occasions. Immediately after the adhesion phase of the microorganisms on the specimens, the biofilm was washed two times with PBS, and 1500 μL of fresh RPMI medium was added to each orifice of the plate, as well as 150 μL of Alamar Blue® solution. The plates were then placed in the orbital shaking incubator at 37°C and 76 rpm. After 4 hours, the first reading was performed, and 24 hours later, the second fluorescence reading was performed. Fluorescence of the specimens was measured using Fluoroskan Ascent at 560 nm (A560) and 590 nm (A590).

### Cytotoxicity assay

After immersion in the solutions according to each experimental group, the specimens (n = 9) were washed in running water to remove excess of disinfectant solutions. Subsequently, the specimens were placed under ultraviolet light for 20 minutes per side for disinfection [29].

To evaluate the cytotoxic effect of the substances incorporated and released by the specimens, eluates from these specimens were obtained [29–33]. For this purpose, three specimens from each experimental group (n = 3) were placed into test tubes with 3 mL of Eagle culture medium and incubated at 37°C for 24 hours [30]. During this period, probably toxic substances were diffused into the culture medium, thus creating the eluates that were used in the cytotoxicity assay. A tube containing only 3 mL of culture medium was stored under the same conditions (negative control group).

The possible cytotoxic effect of the substances released by the resins was evaluated by the cell culture method. Thus, keratinocytes cells (HaCaT 0341) were propagated in the Dulbecco’s Modified Eagle’s Medium (DMEM) with 7.5% fetal bovine serum and 80 μg/mL gentamicin. The culture was maintained at 37°C in an atmosphere of 5% CO_2_ and 95% air. Next, the cells (1 × 10^4^ cell/mL) were seeded into 96-well culture plates and incubated for 24 hours at 37°C in the same atmosphere.

After this period, the culture medium was removed and 50 μL of fresh DEMEM were placed in each well. In addition, 50 μL of the extract containing the substances released by the specimens were placed in each well. The plate was incubated for 24 hours in the incubator with 5% CO_2_ at 37°C. For each experimental group, four wells were used (quadruplicate analysis). Four wells did not receive the eluates and received only 100 μL of the culture medium (negative control group).

### Biofilm reduction assay

The antiseptic soaps were diluted in saline solution at their MIC values. Similar to the biofilm inhibition test, the microbial reactivation, the pre-inoculum, and the inoculum were performed in the same manner as described to determine the MICs.

For the biofilm inhibition analysis, the specimens, after storage for 48 hours in distilled water, were washed in running water and placed in an ultrasonic cube for 20 minutes on each side to remove any residue from the surface. Thereafter, the specimens were placed under UV light for 20 minutes on each side for surface disinfection and were then individually placed in a 24-well plate containing 750 μL of RPMI medium with *C*. *albicans*. After this, the plate was placed in an orbital shaker incubator for 90 minutes at 37°C and 76 rpm for cell adhesion. After this time, the contents of the wells were removed from the plate, and the wells were washed twice with PBS to remove unbound cells. New RPMI medium was added to the wells, and the plate was returned to the incubator for 48 hours for formation of mature biofilm. The RPMI medium was renewed after 24 hours. After 48 hours of biofilm formation, the plate was removed from the incubator and the medium of all wells was discarded. Antiseptic soap solutions (750 μL) were then added to each well, according to the experimental group (n = 9): distilled water, 3.12% Protex, 0.39% Dettol, or 0.78% Lifebuoy. Subsequently, the plate was incubated for an additional 8 hours.

After this time, each well of the plate was washed twice with PBS, and all the specimens were removed from the contaminated wells and transferred to a new plate containing 150 uL of sterile saline in each well. Then, the specimens were scraped with a sterile pipette tip to peel off the formed biofilm. Subsequently, two dilutions of 1:10 were made from this resulting solution. Serial dilutions of 10^−1^ and 10^−2^ were used for seeding Petri dishes containing the SDA culture medium with 5 μg/mL chloramphenicol. After 48 hours of incubation at 37°C, the CFU/mL values were calculated.

### Roughness

The surface roughness of specimens (n = 15) was measured using a profilometer (SJ 400; Mytutoyo Corp). The measurements were performed in the central area of each specimen at intervals of 2.0 mm, and the average reading was designated as the intact Ra value for that specimen. Resolution was 0.01 μm, interval (cutoff length) was 0.8 mm, transverse length was 2.4 mm, and stylus speed was 0.5 mm/second [34]. Three measurements of surface roughness were performed for each specimen, and mean Ra was used for the statistical analysis.

### Hardness

The hardness of the specimens from each group (n = 15) was obtained using a Vickers diamond, which is an effective tool to evaluate the hardness and viscoelastic responses of polymers [35]. A microhardness tester (Micromet 2100) was used, and four measurements were made for each specimen using a time of 10 seconds with a force of 50 grams. The values were tabulated, and the mean was calculated for the statistical analysis.

### Color stability

The color change of the specimens (n = 15) in the different periods was determined using the BYK-Gardner color-guide spectrophometer according to the Commission Internationale de l’Eclairage (CIE) L*a*b* system [36]. The white calibration standard was used as the background for each specimen during data collection to eliminate the effects of differences in background color during measurements. All measurements were repeated twice, and mean for the L*, a*, and b* values was calculated.

The total color change (ΔE) of each specimen was then calculated using the relationship:

$$\Delta E=½\;[{\left(L1-L0\right)}^{2}+{\left(a1-a0\right)}^{2}+{\left(b1-b0\right)}^{2}]$$

To calculate the amount of color change (ΔE) recorded by the spectrophotometer in a clinical environment, the data were converted to National Bureau of Standards (NBS) units (aka NIST, National Institute of Standards and Technology, United States of America) through the equation NBS units = ΔE × 0.92, where critical remarks of color differences are expressed by NBS units (Table 2).

**Table 2:** National Bureau of Standards (NBS) system of expressing color differences.

Critical remark of color difference	NBS* units
**Extremely slight change**	0.0–0.5
**Slight change**	0.5–1.5
**Perceivable change**	1.5–3.0
**Marked change**	3.0–6.0
**Extremely marked change**	6.0–12.0
**Change to another color**	12.0

*Colorimetry National Bureau of Standards Monograph 104; 1968:47.

### Statistical analysis

In order to evaluate if the immersions in the disinfectant solutions interfered in the capacity of the biofilm formation (biofilm inhibition assay) in the different periods (0, 7, 14, 21, and 28 days), two-way analysis of variance (ANOVA) was used, followed by the post-hoc least significant difference (LSD) test. To evaluate if the type of solution significantly reduces *C*. *albicans* biofilm (CFU/mL) after a period of 8 hours, the Kruskal-Wallis nonparametric test was used, followed by Dunn’s post-hoc test, because the assumptions of normal distribution of the dependent variable (CFU/mL) and homoscedasticity of the variances in the different groups tested showed p > 0.05 in the Shapiro-Wilk and Levene tests, respectively.

To evaluate if antiseptic soaps and time significantly influenced the cytotoxicity of a denture base acrylic resin, the parametric two-way ANOVA test was used for independent specimens, followed by the Bonferroni post-hoc test. The assumption of the normal distribution of the dependent variable (cytotoxicity) in the different groups defined by the “soap-type” and “time” factors was evaluated by the Shapiro-Wilk test. For all groups, p > 0.05 was obtained. The assumption of variance homogeneity was evaluated with the Levene test (p = 0.428). In addition, the results were also evaluated in accordance with ISO standard 10993–5 [30], which states that inhibition of < 25% counts as non-cytotoxic, 25–50% as slight, 50–75% as moderate, and > 75% as highly cytotoxic.

To evaluate if the type of soap and time significantly affected the roughness, hardness, and color stability of a denture base acrylic resin, the parametric two-way ANOVA test was used, followed by the Bonferroni post-hoc test. The assumption of the normal distribution of the dependent variable (roughness, hardness, and color stability), homoscedasticity of variances, and sphericity in the different groups defined by the “soap-type” and “time” factors were evaluated by the Shapiro-Wilk, Levene, and Mauchly tests, respectively.

The color change values obtained were also submitted to a qualitative analysis, compared to the standards of the NBS. The system of NBS units was developed to quantify the critical color change value (ΔE) [37].

A significance level of α = 0.05 was used for all inference analyses. Descriptive statistical analyses, graphs, and inferences were performed with the PAWS Statics software (see 19, SPSS Inc., Chicago, IL).

## Results

### Minimum Inhibitory Concentration (MIC)

**Fig 1:**
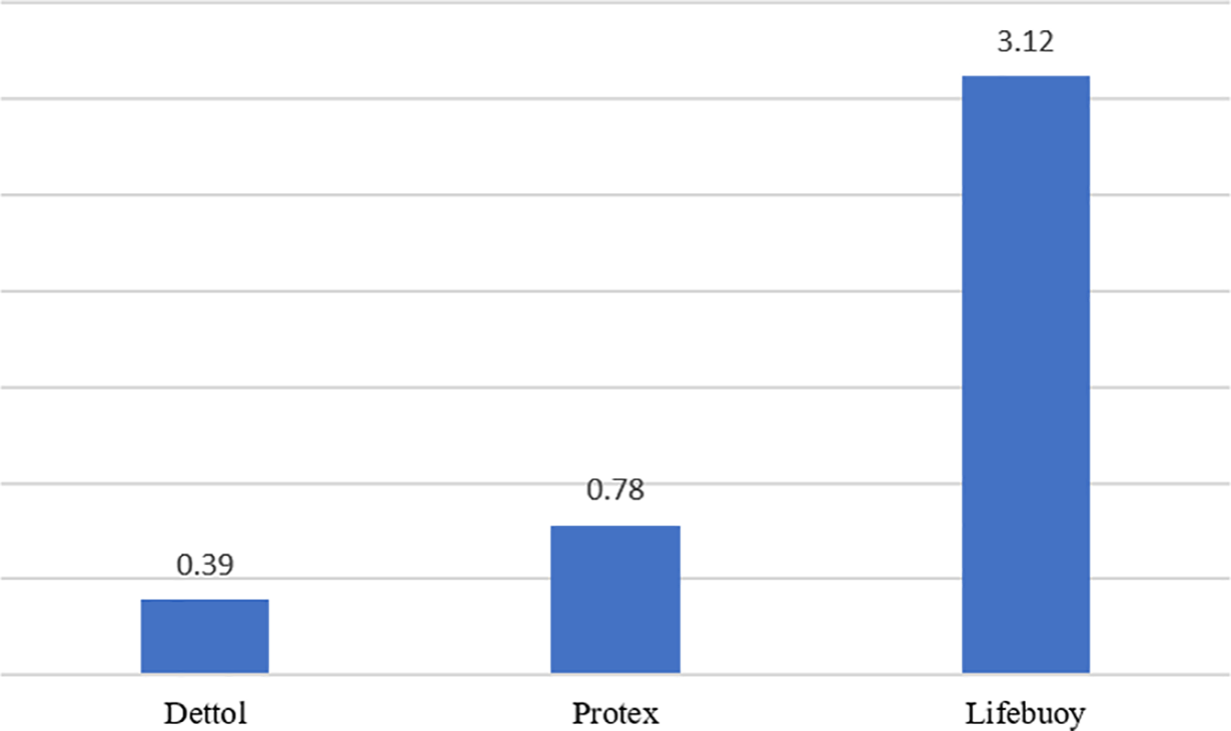
MICs of the antiseptic soaps (%).

The results of the MIC (determined for *Candida albicans* strains) are described in Fig 1. MIC was established as 0.39% for Dettol soap, 3.12% for Protex soap, and 0.78% for Lifebuoy soap.

### pH measurements

In terms of the pH of the dilutions used in this study, the following values were found: distilled water: 7.2; Protex: 5.4; Dettol: 3.9; Lifebuoy: 5.4.

### Biofilm formation capacity

**Fig 2:**
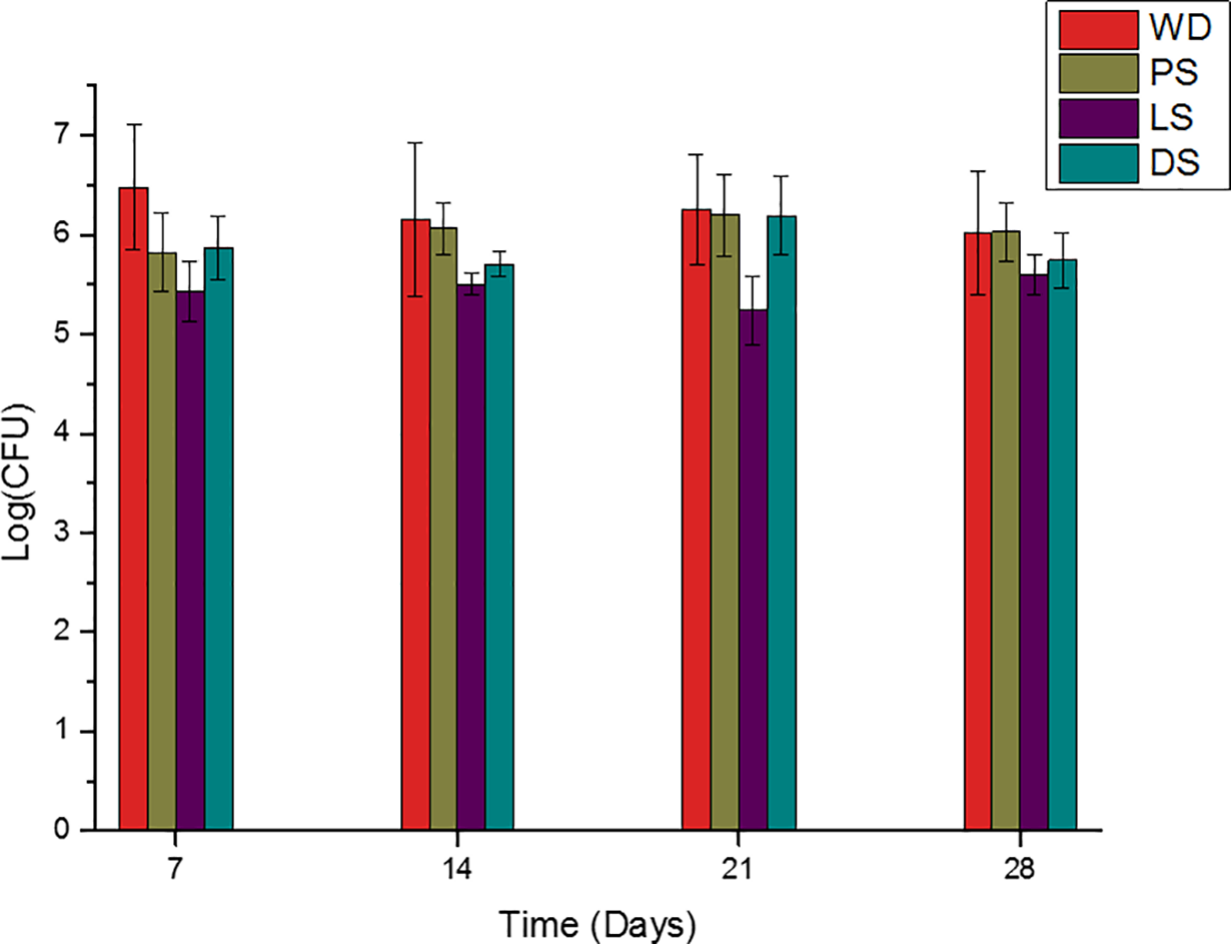
CFU/mL (log) of the antiseptic soaps, according to the immersion period (adhesion phase).

According to the results of CFU in the adhesion phase (time 0), the type of soap had a statistically significant effect (p = 0.02929) on the biofilm formation capacity. However, the time did not have statistically significant effect (p = 0.5757). The time was not influenced by the type of soap (or vice versa), as suggested by the non-significant interaction between the two factors (p = 0.06133). Fig 2 shows the results of the experimental groups in the adhesion phase, where the CFU count is represented in logarithms.

**Fig 3:**
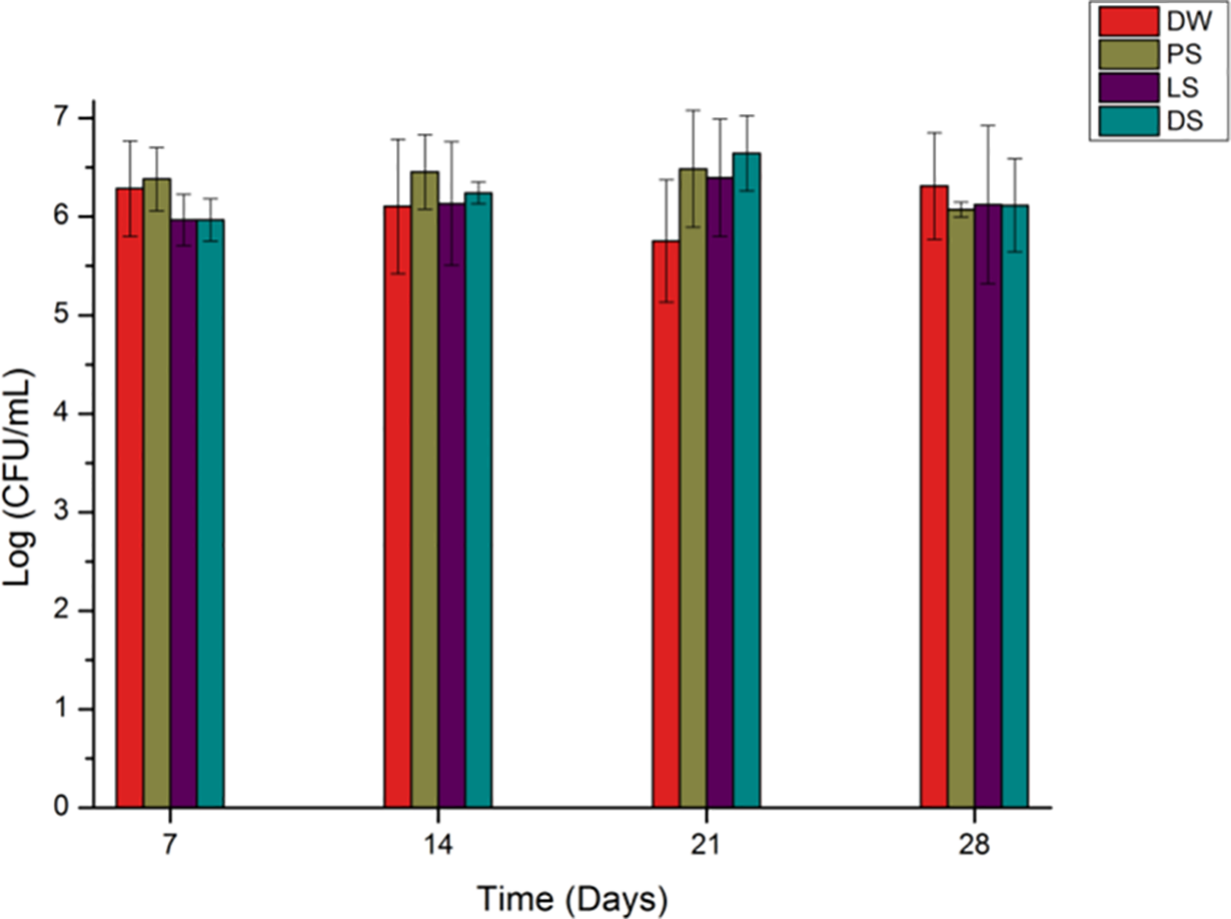
Concentration of CFU/mL (log) of the antiseptic soap, according to the immersion period (24 hours).

According to the results of CFU after 24 hours (biofilm), the type of soap and storage did not have a statistically significant effect on the biofilm formation capacity (p = 0.10802 and p = 0.35087, respectively). In addition, the time was not influenced by the type of soap (or vice versa), as suggested by the non-significant interaction between the two factors (p = 0.58241). Fig 3 shows the results of the experiment after 24 hours, where the count of CFU is represented in logarithms.

**Fig 4:**
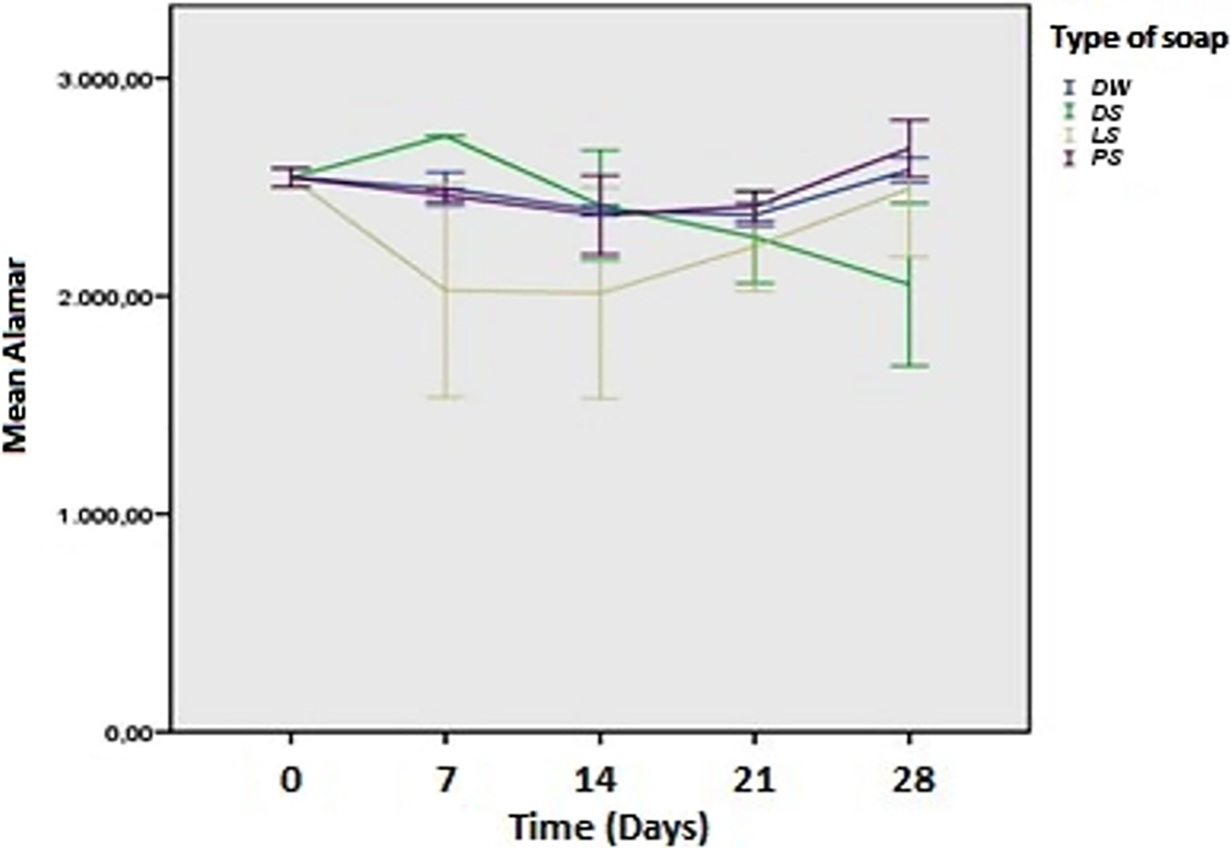
Fluorescence values in the adhesion phase depending on the type of soap and storage time.

The cell proliferation results from the Alamar Blue assay for the adhesion phase (time zero) showed that the type of soap (p = 0.022) had a statistically significant effect (p < 0.05) on the capacity of the biofilm formation, which did not occur with storage time (p = 0.052). Moreover, according to the results, it is possible to affirm that the interaction of the type of soap with the time (p = 0.018) had a statistically significant effect (p < 0.05) on the capacity of biofilm formation on the acrylic resin. Fig 4 shows the fluorescence values in the adhesion phase as a function of the type of soap and storage time.

**Fig 5:**
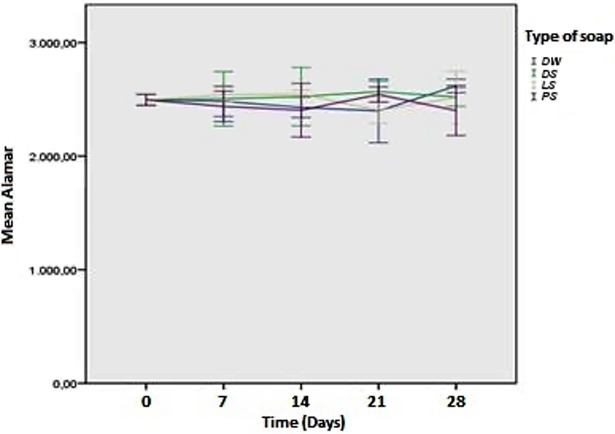
Fluorescence values after 24 hours of biofilm formation depending on the type of soap and storage time.

Based on the Alamar Blue assay results after 24 hours of biofilm formation, the type of soap (p = 0.674), storage time (p = 0.973), and interaction of the type of soap with the storage time (p = 0.812) did not have a statistically significant effect (p > 0.05) on the biofilm formation capacity on the acrylic resin after 24 hours. Fig 5 shows the fluorescence values after 24 hours of biofilm formation as a function of the type of soap and storage time.

### Cytotoxicity assay

**Fig 6:**
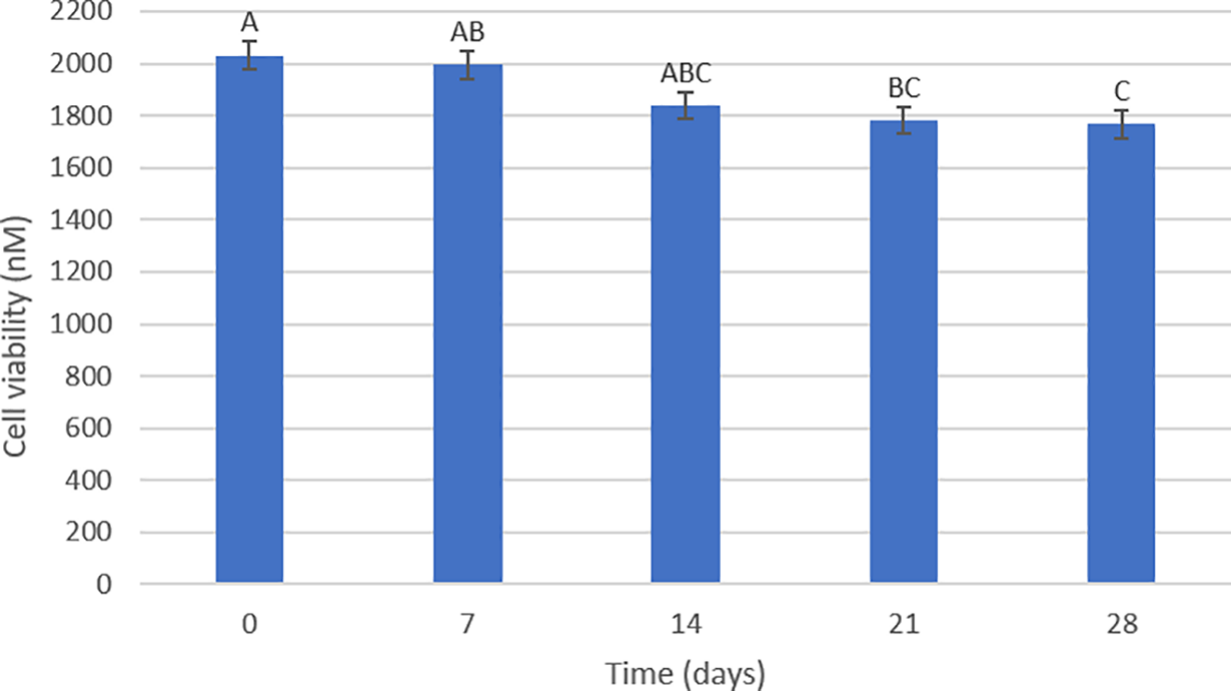
Cell viability in relation to time. The bars with different letters represent statistically significant differences according to the Bonferroni test (p < 0.05).

**Fig 7:**
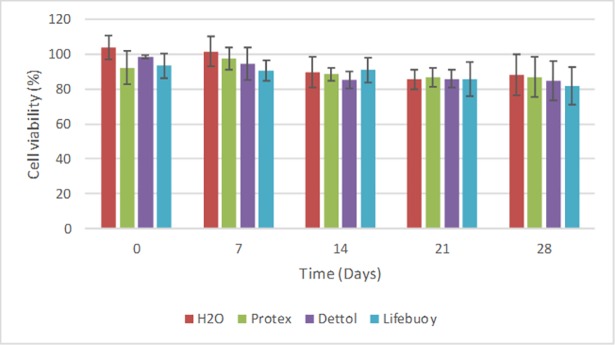
Cell viability of the disinfectant soaps according to the control group (%).

According to the results of the two-way ANOVA test regarding the cytotoxicity of the samples after immersion in the antiseptic soaps, the type of soap (p = 0.154) did not have a statistically significant effect. However, the time (p = 0.001) had a statistically significant effect (p < 0.05) on the cytotoxicity of the acrylic resin. After a period of 21 and 28 days of immersion in the solutions (Fig 6), the resin showed the lowest values of cell viability (μ = 1782.9 ± 52.5 and μ = 1766.8 ± 52.5, respectively) with no statistically significant difference between these times. However, when compared with the 0-day period (μ = 2031.3 ± 52.5) both groups presented a statistically significant difference. Finally, the effect of time on cytotoxicity was not influenced by the type of soap (or vice versa), as suggested by the non-significant interaction between the two factors (p = 0.919). Fig 7 illustrates the results of the quantitative analysis. It was possible to observe that the acrylic resin, after immersion in the soaps, was classified as non-cytotoxic because it presented inhibition of less than 25% in relation to the control group, regardless of storage time.

### Biofilm inhibition assay

**Fig 8:**
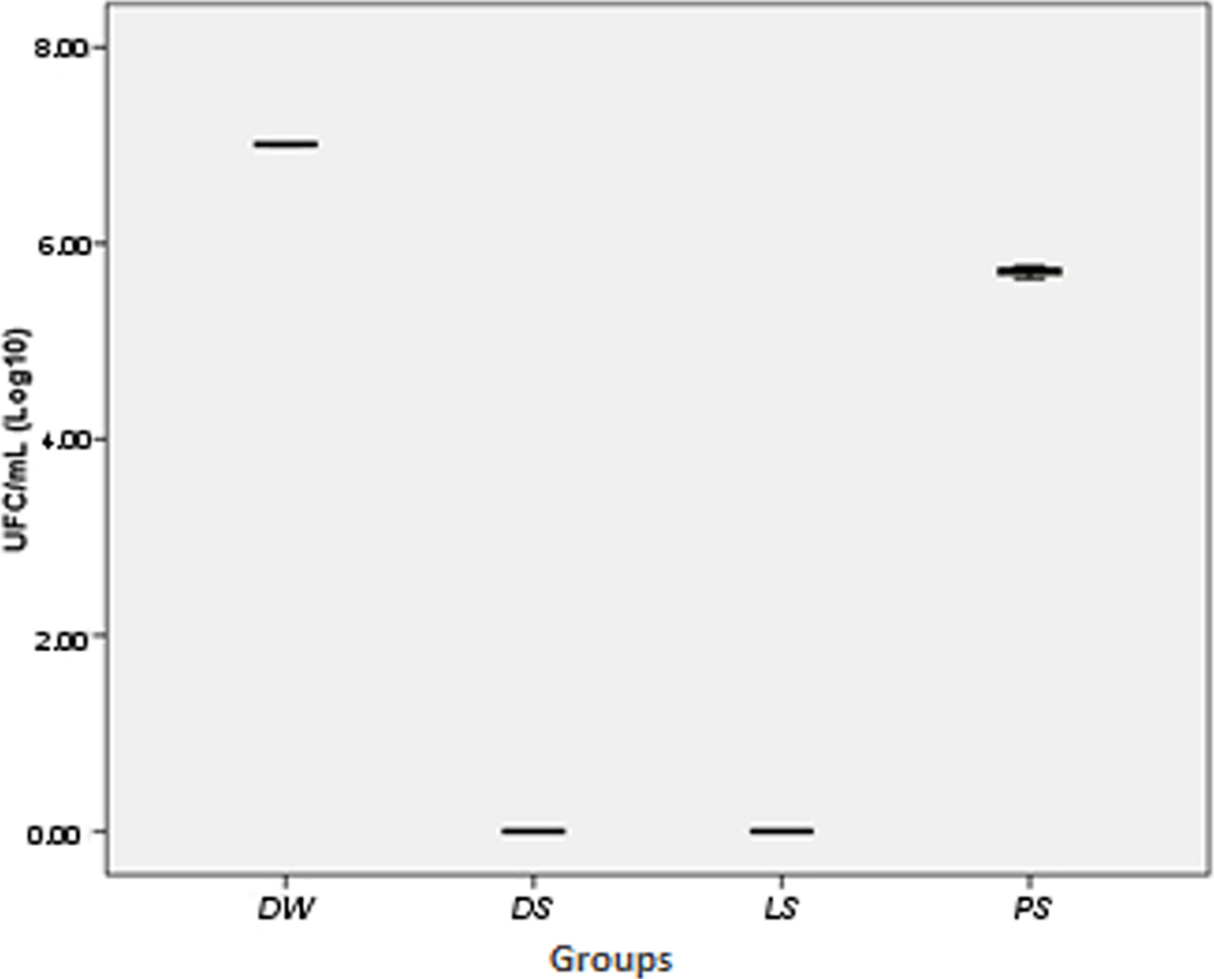
Box-plot distribution of the data of the different groups tested for the evaluation of colony-forming units (CFU/mL).

The Kruskal-Wallis test showed a statistically significant difference (p = 0.014) when the different groups were compared. Table 3 shows the Dunn multiple comparisons between the different tested groups. Table 4 shows the average of the different groups tested. Fig 8 shows the distribution of the data of the different groups tested. In summary, the results show that all the solutions were effective when the ability to reduce the biofilm on the surface of the samples of acrylic resin after 8 hours of immersion was tested. Dettol and Lifebuoy totally eliminated the formed biofilm.

**Table 3:** Dunn’s multiple comparisons among different experimental groups.

Comparison by groups	P
Dettol—Lifebuoy	1.000
Dettol—Protex	0.616
Dettol—Control	0.039
Lifebuoy—Protex	0.616
Lifebuoy—Control	0.039
Protex—Control	1.000

**Table 4:** Average of the different tested groups.

	Groups	N	Mean
**8 Hours**	Control	3	11.00
	Dettol Mic	3	3.50
	Lifebuoy Mic	3	3.50
	Protex Mic	3	8.00
	Total	12	

### Roughness

According to the results of the two-way ANOVA test for the roughness values, there was no statistically significant difference for the main effects (p = 0.209) and the interaction (group and time, p = 0.989).

### Hardness

According to the results of the two-way ANOVA test for the hardness values, the type of soap (p = 0.003) and storage time (p < 0.001) had a statistically significant effect (p < 0.05) on the hardness of the denture base acrylic resin. However, the effect of time on hardness was not influenced by the type of soap (or vice versa), as suggested by the non-significant interaction between the two factors (p = 0.107).

**Fig 9:**
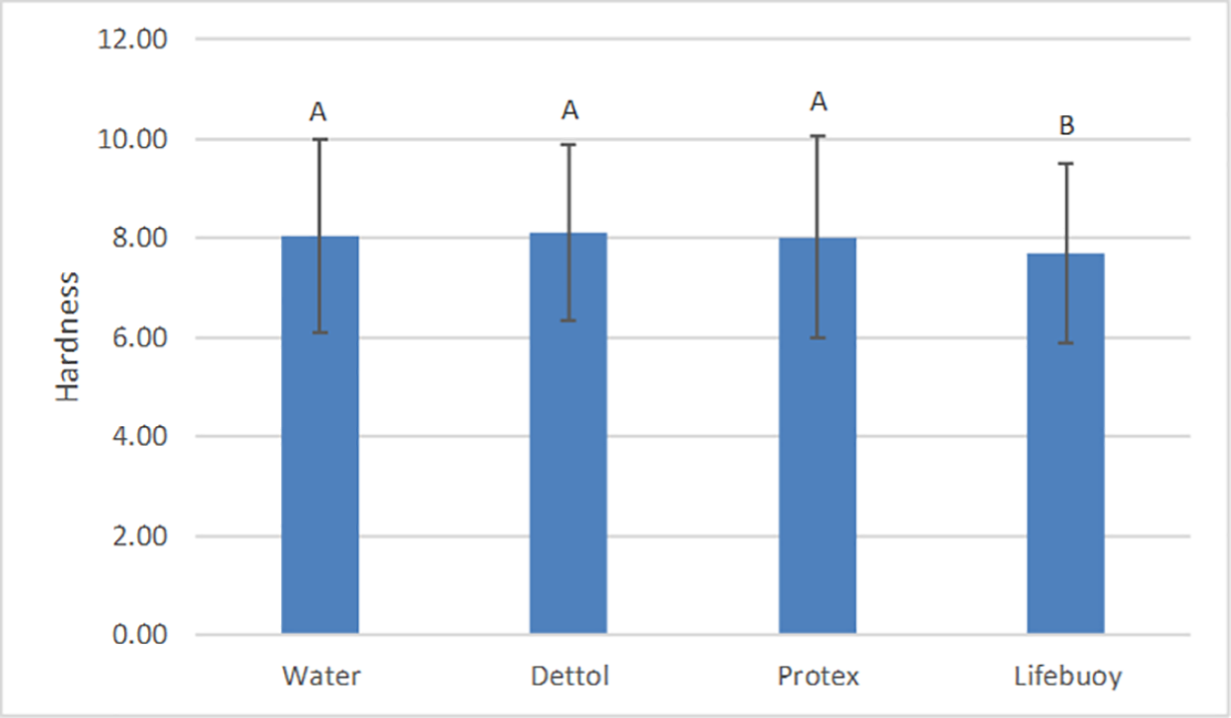
Hardness compared to the type of soap. The bars with different letters represent statistically significant differences according to the Bonferroni test (p < 0.05).

**Fig 10:**
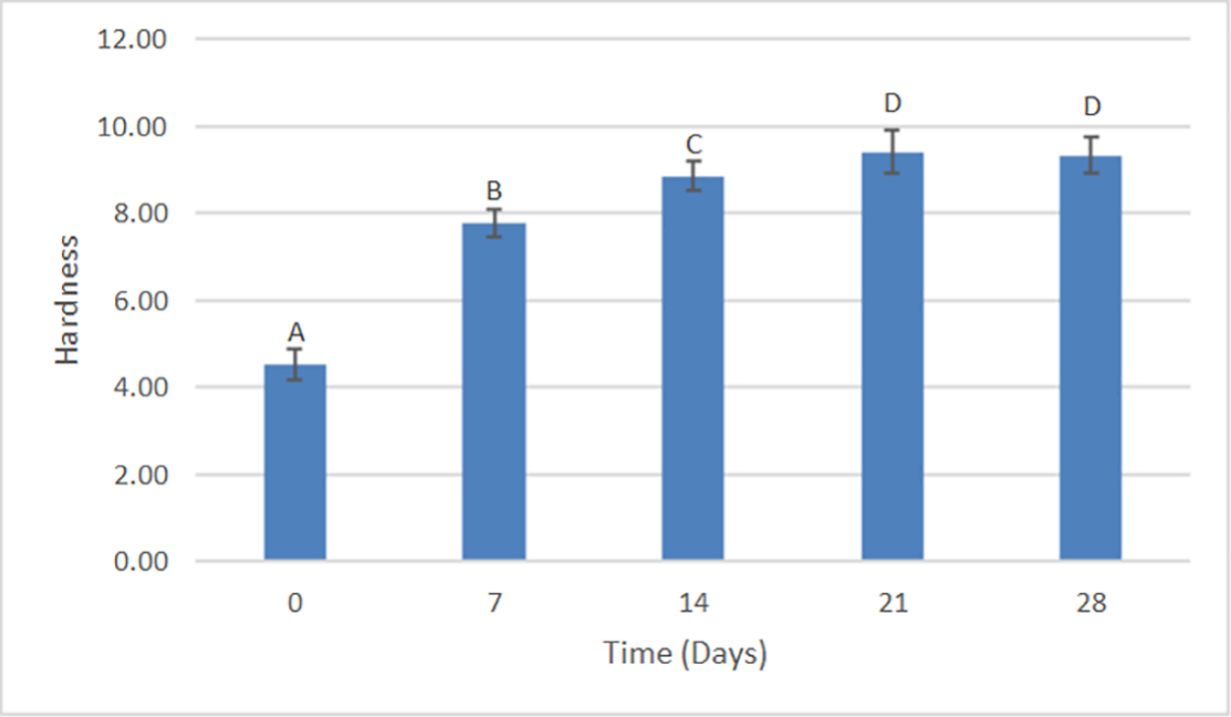
Hardness in relation to time. The bars with different letters represent statistically significant differences according to the Bonferroni test (p < 0.05).

From the analysis of Fig 9, it can be observed that the LS group (μ = 7.70 ± 1.82) presented the lowest hardness values, being statistically different when compared to the other study groups: DW (μ = 8.04 ± 1.95), DS (μ = 8.13 ± 1.77), and PS (μ = 8.02 ± 1.82). Fig 10 shows that after 21 and 28 days, the resin had the highest values of hardness (μ = 9.41 ± 0.49 and μ = 9.33 ± 0.42, respectively), with no statistically significant difference at these times. However, when compared to the periods of 0 (μ = 4.51 ± 0.35), 7 (μ = 7.77 ± 0.30), and 14 (μ = 8.85 ± 0.49) days, both groups (21 and 28 days) presented a statistically significant difference.

### Color stability

The results of the two-way ANOVA test for the color change values showed that the type of soap (p = 0.003) and storage time (p = 0.045) had a statistically significant effect (p < 0.05) on the color change of the denture base acrylic resin. The effect of time on color stability was not influenced by the type of soap (or vice versa), as suggested by the non-significant interaction between the two factors (p = 0.088).

**Fig 11:**
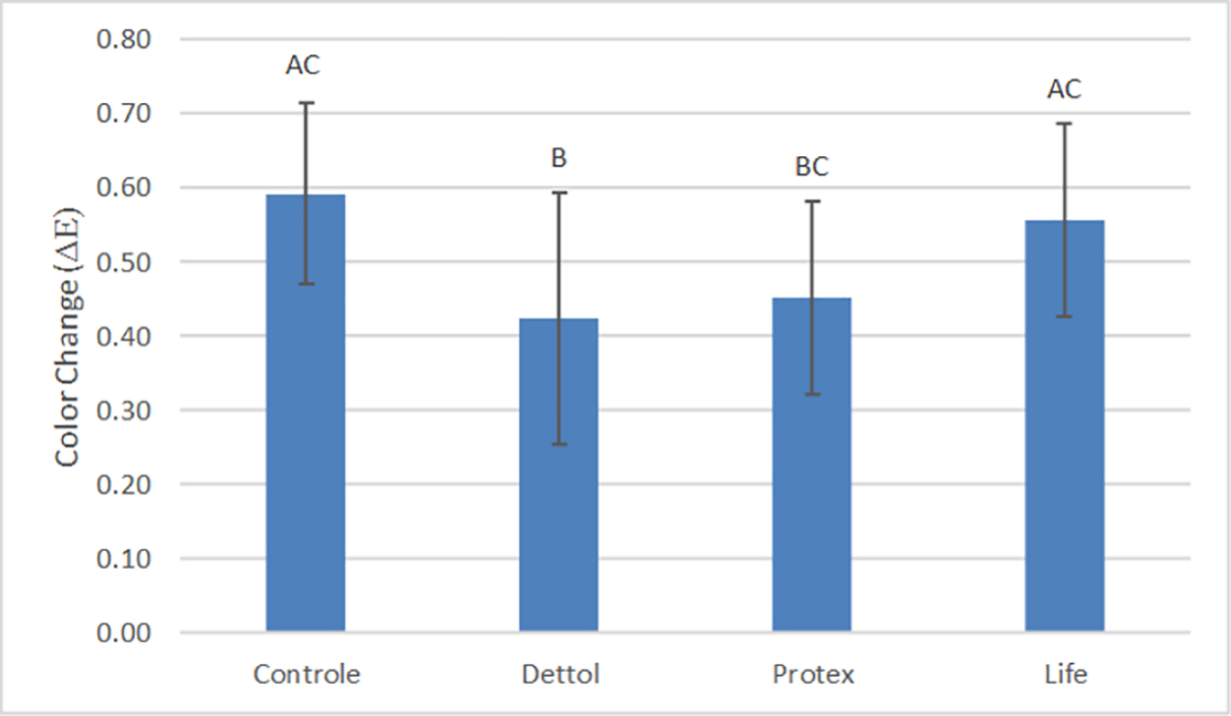
Color change relative to the type of soap. The bars with different letters represent statistically significant differences according to the Bonferroni test (p < 0.05).

Fig 11 shows the color change relative to the type of soap. The bars with different letters represent statistically significant differences according to the Bonferroni test (p < 0.05). Statistically significant differences for this factor occurred between the DW and DS groups (p = 0.007) and the DW and PS groups (p = 0.029). The DW (μ = 0.59 ± 0.12) and LS groups (μ = 0.56 ± 0.12) presented the highest values of color change, followed by the PS (μ = 0.45 ± 0.10) and DS groups (μ = 0.42 ± 0.15), which were statistically equal to each other.

**Fig 12:**
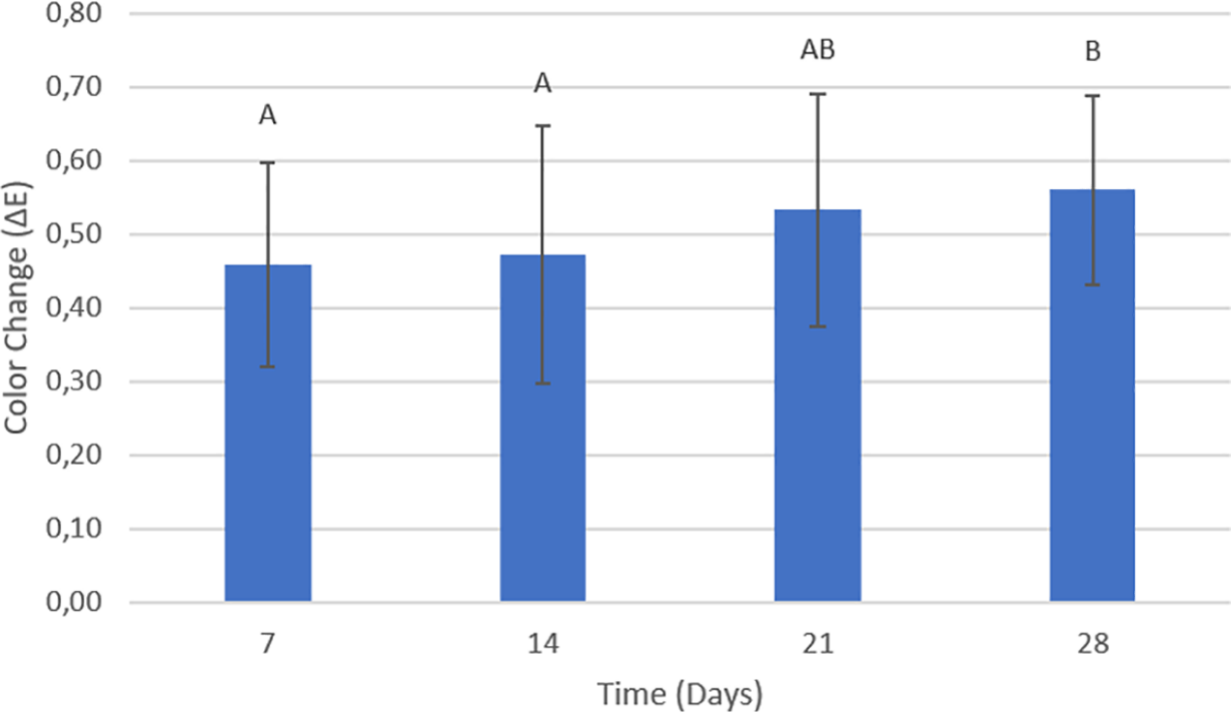
Color change over time. The bars with different letters represent statistically significant differences according to the LSD test (p < 0.05).

The color change with respect to time is shown in Fig 12. The bars with different letters represent a statistically significant difference according to the LSD test (p < 0.05). After 28 days, the acrylic resin presented the highest values of color change when compared with the 7- and 14-day periods, being statistically significant (p = 0.032 and p = 0.023, respectively). However, there was no significant difference when compared to the 21-day period (p = 0.517). Finally, the effect of time on color change was not influenced by the type of soap (or vice versa), as suggested by the non-significant interaction between the two factors (p = 0.088).

According to the qualitative analysis of the results of this study (Table 5), all values of NBS units were between 0.27 and 0.58, indicating extremely slight or slight changes for all groups.

**Table 5:** Clinical Relevance (NBS units).

	Groups	Mean	NBS
**7 days**	DW	0.52	0.48
	DS	0.48	0.44
	PS	0.32	0.30
	LS	0.50	0.46
**14 days**	DW	0.62	0.58
	DS	0.29	0.27
	PS	0.43	0.40
	LS	0.53	0.49
**21 days**	DW	0.63	0.59
	DS	0.37	0.35
	PS	0.49	0.45
	LS	0.62	0.57
**28 days**	DW	0.57	0.53
	DS	0.54	0.50
	PS	0.55	0.51
	LS	0.57	0.53

Statistical analysis can be found in the Supporting Information file (S1 Table: Tables A-J).

## Discussion

A variety of methods have been proposed for the disinfection of denture surfaces, including the use of disinfectant solutions, antiseptic mouthwashes, microwave disinfection, and, recently, antimicrobial photodynamic therapy. However, studies have shown some disadvantages regarding the use of these methods [18–21]. In addition, besides being an expensive method, the role of antimicrobial photodynamic therapy in the disinfection of acrylic resin surfaces is unclear [38]. The results of this study present a low-cost, easy-access procedure with high potential to become a disinfection protocol for removable partial or total dentures, which can be used for the aging patient population. Furthermore, there are no reports in the literature for this purpose. The null hypothesis of this study was partially accepted.

During the adhesion phase, for cell counts and assessment of cellular proliferation, biofilm formation capacity decreased during the first days of storage when the samples were immersed in the Lifebouy solution; however, it increased after 28 days of storage. Lifebouy, unlike other soaps, presents, in its composition, linalool (open-chain monoterpene alcohol), a natural compound that manifests strong antifungal activity *in vitro* [39]. Regarding the 24-hour biofilm, in both assays (CFU and Alamar Blue), the results showed no difference between solutions, with biofilm formation having occurred in all groups. Furthermore, storage time did not influence the capacity of biofilm formation. Storage of samples in solutions for long periods of time, especially in acidic solutions, may have favored superficial deterioration of the acrylic resin samples, increasing their surface areas. Superficial deterioration can form niches that act as microorganism reservoirs, stimulating adhesion and proliferation of fungal cells.

In terms of biofilm reduction capacity after 8 hours of immersion in disinfectant solutions (overnight immersion), the group immersed in Protex soap presented slight biofilm reduction compared to the control group (distilled water), and the groups immersed in Dettol and Lifebouy presented total elimination of biofilm on the surfaces of the samples. This fact may be due to the composition of these materials and the concentration of their active components. Among the active components present in the soaps used are the following: EDTA (ethylenediaminetetraacetic acid), cocamidopropyl betaine, sodium hydroxide, citric acid, and triclocarban (which presents active mechanisms similar to triclosan). Finnegan and Percival [40] reported the capacity of EDTA in destabilizing biofilms through its calcium, magnesium, zinc, and iron sequestration activity, making it an adequate agent for biofilm inhibition. Nowadays, EDTA, tEDTA (tetrasodium EDTA) more specifically, is being explored as an antimicrobial and homogenous antibiotic agent because of its synergic combination capacity with other antimicrobials, leading to a multidirectional approach to biofilm control [40]. Additionally, tEDTA reduced the biofilm viability of salivary bacteria and of *Candida albicans* on the denture base in more than 99% [41]. Cocamidopropyl betaine is a cleaning agent used to treat infected wounds. Minnich et al. [42] performed an *in vitro* study to examine the antimicrobial effects of a solution containing 0.1% betaine. The researchers observed reductions of 5.3 log to 5.8 log for various microorganisms, among them *Candida albicans*. Sodium hydroxide is able to neutralize amino acids and degrade fatty acids, acting on the microorganism cell membrane, which provides its antimicrobial property [43]. Its presence in Dettol and Lifebouy soaps can explain why their solutions achieved better biofilm elimination results than Protex after 8 hours of immersion. Citric acid has been used as a denture disinfectant because of its proven efficiency in reducing the cellular viability of *Candida albicans* in mature biofilm [44]. Dettol was the only product used in this experiment that had citric acid in its composition, which may have been a strong contributor to the positive results obtained. The antiseptic agent triclocarban, present in Protex soap, manifests an ample spectrum [45], making it effective against fungus and gram-negative and gram-positive bacteria. At low concentrations, observed in commercial products, it presents good tolerance for skin and buccal cavity use [46]. In these conditions, it is an effective bacteriostatic agent, which inhibits the synthesis of fatty acids. At high concentrations, triclocarban acts as a biocide, which contains multiple cytoplasmic and membrane targets [46]. Triclocarban easily crosses cell membranes, and, once inside the cell, it acts on a specific enzyme that is vital to the survival of the bacteria and fungus [46]. The soap manufacturers do not reveal the concentrations of the components that make up their products. Therefore, the discussion regarding the results obtained in this study is largely based on assumptions. Identification of the components present in each soap and their respective concentrations is necessary to expand this research.

Regarding cytotoxicity, the results obtained in this study show that there was no significant difference in cell viability between the samples immersed in soap solutions or water. Therefore, at first, the use of these solutions for prosthesis disinfection in night immersion would not have a cytotoxic effect on oral mucosal cells. However, immersion time significantly influenced the cytotoxicity of acrylic resins. After 21 days, there was a decrease in cell metabolism due to prolonged immersion time, regardless of which solution was being analyzed. The increase of cytotoxicity does not seem to be related to direct actions of the soap components on the cells because the group immersed in distilled water also presented this increase. A clinically significant consequence of acrylic resin biodegradation is the release of monomers and toxic additives not connected to polymer networks [47]. The compounds released can have a toxic effect in the oral cavity. Cell-based studies indicate that these products, which emerge from acrylic resin biodegradation (methyl methacrylate and its derivatives, phthalates, formaldehyde), have the potential to induce cytotoxicity, genotoxicity, alterations in the expression of cytokines, growth factors, and oxidative stress in primary and permanent cells [29,48–50]. However, through qualitative analysis of data, acrylic resins, after immersion in soaps, were proven not to be cytotoxic because they presented inhibition rates lower than 25% (regardless of the storage time) when compared to the control group.

Literature shows that the physical properties of denture base acrylic resins may be altered when constantly exposed to liquids, such as drinks and mouthwashes [51], because, when placed in aqueous environments, the acrylic resins are capable of absorbing a small quantity of water through diffusion. These water molecules penetrate the resin matrix and position themselves between the polymer chains, causing a slight increase in volume, which interferes with the chain entanglement and alters the physical characteristics of the polymer [52]. These facts may be associated with the increase of monomer liberation by the material as time advances, resulting in property alterations as well as an increase in cytotoxicity. Additionally, the acidity of the solutions associated with the solubility of acrylic resins in low pH may modify the connectivity of the polymers over time and make them more susceptible to degradation. This phenomenon can negatively affect resistance to wear, roughness, and superficial integrity due to matrix softening [53,54], which permits us to conclude that provoking alterations in their physical properties can, consequently, also affect biological properties.

Reports suggest a relationship between superficial roughness and *Candida albicans* adhesion to acrylic resins that compose dental prostheses [7,55]. The results of this study demonstrated that roughness did not change after immersion in the solutions, which is in agreement with the results obtained by Machado et al. [56]. The stability of the material could be due to its reticulated polymeric structure formed from a thermal polymeric reaction, in which a high rate of monomers converts into polymers, making the material more stable [57]. On the other hand, some studies have reported surface roughness alterations on resin samples after disinfection processes [15,58]. The difference between these results may be explained by the type of solutions used. The present study was the first to propose disinfection of prostheses using immersion in liquid disinfectant soaps, making it difficult to directly compare our results to those obtained in other studies.

Regarding hardness, only the samples immersed in Lifebouy soap presented low hardness values, being statistically different than the other groups analyzed. The difference between the results obtained from the different solutions may be explained by the composition of the disinfectant soaps. Some of the components can act as solvents on the resins’ surfaces, considered potential sources of damage [59]. As stated previously, the hardness results remained unaltered for most of the solutions. These results agree with other studies [58, 60]. Regarding storage time, the highest values of hardness were found after 21 and 28 days. The results of the present study agree with the results obtained by Neppelenbroek et al. [59]. However, they conflict with the results obtained by Panariello et al. [24] and Goiato et al. [60]. This contrast can be explained by the different storage periods because, in the present study, the samples were immersed for a maximum period of 28 days. At first, the water molecules, when absorbed by the resin, act as plasticizers, resulting in linking cleavage and gradual infrastructure degradation [14]. Additionally, the monomer content can adversely affect the physical properties of the prosthesis due to the plasticizing effect right after polymerization. When stored for long periods of time, the release of residual monomers from the interior on the polymeric materials can contribute to the increase of hardness values [59].

In the present study, the samples immersed in distilled water and in Lifebouy presented the highest color alterations, followed by the groups immersed in Protex and Dettol. The main mechanism responsible for color alteration is liquid absorbance. When the resin absorbs water, its polymeric matrix expands, separating the polymeric chains, which stains or discolors the material. When water molecules are absorbed by the resin, they act as plasticizers, causing link cleavage and gradual infrastructure deterioration [14]. Considering this information, color alterations can be explained by the absorbance of water, component dissolution, and intrinsic pigment degradation of resins [60,61]. Additionally, the medium’s pH may have influenced the discoloration of the resin because acidity can make surfaces rougher, favoring the occurrence of stains [62]. Another important factor to consider is that alcohol can also favor staining because it causes softening of the resin’s matrix. The pH and chemical compositions of the different disinfectant soaps can explain the difference detected regarding color stability. However, the mechanisms by which the color alterations occur could not be exactly known; therefore, they were only estimated. The results described above agree with the findings of Panariello et al. [24] and Fernandes et al. [63]. Regarding time, the results showed that after the 28-day period, color alterations were higher than after 7 and 14 days. Similar results were detected in studies conducted by Panariello et al. [24] and Goiato et al. [60]. According to Moffa et al. [11], color stability of dental material is a variable that should be considered when choosing an adequate hygiene method, although time would ultimately have an important impact, regardless of the disinfectant chemical products used. In order to standardize the results of different studies, the NBS unit system is commonly used. This system allows ΔE data to be converted into clinically relevant results. The analysis of the results of this study verified that all color alterations were between 0.27 and 0.58 NBS units, indicating imperceptible or slight alterations for all groups.

According to the results obtained in the present study, liquid soaps can be an alternative for removable denture hygiene, taking into consideration their effectiveness in reducing biofilm and their lack of cytotoxicity. Roughness, hardness, and color alteration results should also be considered after immersion in disinfection solutions, which, in general, presented the same behaviors of acrylic resin immersion in distilled water. Despite the favorable results, there are some limitations to the study, such as not including a positive control group, use of single cultures, and short-term follow-up. In addition, other studies must be conducted, including clinical studies, in order to establish a disinfection protocol, which can then be used by patients.

## Conclusion

For all the antiseptic soaps, MICs for *Candida albicans* were found at high dilutions, which demonstrates their efficacy. Although the results of the tests of biofilm formation capacity after different immersion periods did not present significant differences, and although biofilm formation was observed for all groups, all the solutions were effective in terms of the ability to reduce the biofilm of the surface of the acrylic resin samples after 8 hours of immersion. Dettol and Lifebuoy totally eliminated the formed biofilm. Another positive result of this study was the classification of the three soaps as non-cytotoxic. There was no change in the roughness values for any of them. However, the Lifebuoy soap changed the hardness and color values of the acrylic resin, regardless of the storage time. These results present an alternative procedure that should be studied further but that has high potential to become a disinfection protocol for removable partial or total dentures.

## Supporting information

S1 Table Tables from statistical analysis. Table A. Two-Way ANOVA for Biofilm Formation Capacity (adhesion phase).
Table B. Two-way ANOVA for Biofilm Formation Capacity (24 hours).
Table C. Two-Way ANOVA for the Alamar Blue assay in the adhesion phase.
Table D. Two-factor ANOVA test for the Alamar Blue assay after 24 hours of biofilm formation.
Table E. Two-way ANOVA for cytotoxicity assay.
Table F. Two-way ANOVA for roughness.
Table G. Means and the standard deviation of roughness for all groups evaluated.
Table H. Two-way ANOVA for hardness.
Table I. Two-way ANOVA for color stability.
Table J. Means and the standard deviation of color stability for all groups evaluated.
(DOCX)
