# Supplementary material for: Properties of an acrylic resin after immersion in antiseptic soaps: Low-cost, easy-access procedure for the prevention of denture stomatitis
Source: PLoS One. 2018 Aug 30;13(8):e0203187. doi: 10.1371/journal.pone.0203187 (PMC6117035; doi:10.1371/journal.pone.0203187)
Supplement: S1 Table — Table A. Two-Way ANOVA for Biofilm Formation Capacity (adhesion phase). Table B. Two-way ANOVA for Biofilm Formation Capacity (24 hours). Table C. Two-Way ANOVA for the Alamar Blue assay in the adhesion phase. Table D. Two-factor ANOVA test for the Alamar Blue assay after 24 hours of biofilm formation. Table E. Two-way ANOVA for cytotoxicity assay. Table F. Two-way ANOVA for roughness. Table G. Means and the standard deviation of roughness for all groups evaluated. Table H. Two-way ANOVA for hardness. Table I. Two-way ANOVA for color stability. Table J. Means and the standard deviation of color stability for all groups evaluated. (DOCX) [file pone.0203187.s001.docx]

**Table A. Two-Way ANOVA for Biofilm Formation Capacity (adhesion phase).**

|  | **DF** | **Sum of**  **Squares** | **Squares**  **Mean** | **F Value** | **P Value** |
| --- | --- | --- | --- | --- | --- |
| **Solution** | 3 | 244.852 | 0.81617 | 309.551 | 0.02929 |
| **Days** | 3 | 0.52518 | 0.17506 | 0.66395 | 0.5757 |
| **Interaction** | 9 | 444.901 | 0.49433 | 187.487 | 0.06133 |
| **Model** | 15 | 74.227 | 0.49485 | 187.681 | 0.03125 |
| **Error** | 128 | 3.374.896 | 0.26366 | -- | -- |
| **Total** | 143 | 4.117.166 | -- | -- | -- |

**Table B. Two-way ANOVA for Biofilm Formation Capacity (24 hours).**

|  | **DF** | **Sum of Squares** | **Squares**  **Mean** | **F Value** | **P Value** |
| --- | --- | --- | --- | --- | --- |
| **Solution** | 3.00E+00 | 1.15E+12 | 384.353.000.000 | 206.557 | 0.10802 |
| **Days** | 3.00E+00 | 6.15E+11 | 205.068.000.000 | 110.207 | 0.35087 |
| **Interaction** | 9.00E+00 | 1.40E+12 | 1.56E+11 | 0.8381 | 0.58241 |
| **Model** | 1.50E+01 | 3.17E+12 | 211.455.000.000 | 113.639 | 0.33108 |
| **Error** | 1.28E+02 | 2.38E+13 | 1.86E+11 | -- | -- |
| **Total** | 1.43E+02 | 2.70E+13 | -- | -- | -- |

**Table C. Two-Way ANOVA for the Alamar Blue assay in the adhesion phase.**

|  | **Sum of**  **Squares** | **Df** | **Middle Square** | **F** | **P** | **Power rating** |
| --- | --- | --- | --- | --- | --- | --- |
| **Type of soap** | 502695.312 | 3 | 167565.104 | 3.579 | 0.022 | 0.749 |
| **Time** | 483794.558 | 4 | 120948.640 | 2.584 | 0.052 | 0.674 |
| **Soap x time** | 1356434.875 | 12 | 113036.240 | 2.415 | 0.018 | 0.913 |
| **Error** | 1872563.722 | 40 | 46814.093 |  |  |  |
| **Total** | 352366578.972 | 60 |  |  |  |  |

**Table D. Two-factor ANOVA test for the Alamar Blue assay after 24 hours of biofilm formation.**

|  | **Sum of Squares** | **Df** | **Middle Square** | **F** | **P** |
| --- | --- | --- | --- | --- | --- |
| **Type of soap** | 35155.622 | 3 | 11718.541 | 0.516 | 0.674 |
| **Time** | 11203.159 | 4 | 2800.790 | 0.123 | 0.973 |
| **Soap x time** | 169209.063 | 12 | 14100.755 | 0.620 | 0.812 |
| **Error** | 908995.037 | 40 | 22724.876 |  |  |
| **Total** | 373326468.667 | 60 |  |  |  |

**Table E. Two-way ANOVA for cytotoxicity assay.**

|  | **Sum of Squares** | **Middle Square** | **F** | **P** |
| --- | --- | --- | --- | --- |
| **Type of soap** | 183345.750 | 61115.250 | 1.848 | 0.154 |
| **Time** | 717662.327 | 179415.582 | 5.424 | 0.001* |
| **Soap x time** | 187569.490 | 15630.791 | 0.473 | 0.919 |
| **Error** | 1323011.792 | 33075.295 |  |  |

**Table F. Two-way ANOVA for roughness.**

| **Roughness** | | | | |
| --- | --- | --- | --- | --- |
| **Effect** | ***df*** | **Mean** | **F** | **Value p** |
| **Between groups** |  |  |  |  |
| Group | 3 | 0.063 | 1.689 | 0.209 |
| Residue | 16 | 0.037 |  |  |
| **Within Groups** |  |  |  |  |
| Time | 4 | 0.063 | 1.779 | 0.144 |
| Group * Time | 12 | 0.010 | 0.289 | 0.989 |
| Residue | 64 | 0.035 |  |  |
| Significant at 5% level |  |  |  |  |

**Table G. Means and the standard deviation of roughness for all groups evaluated.**

|  | **Groups** | **Mean** | **Standard deviation** |
| --- | --- | --- | --- |
| **0 days** | DW | 3.2864 | 0.21804 |
|  | DS | 3.3178 | 0.29466 |
|  | PS | 3.3100 | 0.17308 |
|  | LS | 3.2556 | 0.21502 |
|  | Total | 3.2924 | 0.21200 |
| **7 days** | DW | 3.1891 | 0.16131 |
|  | DS | 3.2533 | 0.28638 |
|  | PS | 3.3282 | 0.11535 |
|  | LS | 3.1296 | 0.14367 |
|  | Total | 3.2251 | 0.18879 |
| **14 days** | DW | 3.3189 | 0.33680 |
|  | DS | 3.4544 | 0.13338 |
|  | PS | 3.3136 | 0.10891 |
|  | LS | 3.3096 | 0.17792 |
|  | Total | 3.3491 | 0.20172 |
| **21 days** | DW | 3.3438 | 0.10829 |
|  | DS | 3.4236 | 0.22207 |
|  | PS | 3.3991 | 0.10091 |
|  | LS | 3.2891 | 0.18173 |
|  | Total | 3.3639 | 0.15744 |
| **28 days** | DW | 3.3064 | 0.08202 |
|  | DS | 3.3560 | 0.19833 |
|  | PS | 3.2282 | 0.13255 |
|  | LS | 3.2233 | 0.14214 |
|  | Total | 3.2785 | 0.14457 |

**Table H. Two-way ANOVA for hardness.**

| **Effect** | ***df*** | **Mean** | **F** | **Value p** |
| --- | --- | --- | --- | --- |
| **Between groups** |  |  |  |  |
| Groups | 3 | 0.885 | 6.978 | 0.003* |
| Residue | 16 | 0.127 |  |  |
| **Within Groups** |  |  |  |  |
| Time | 4 | 83.508 | 747.249 | 0.000* |
| Groups*Time | 12 | 0.181 | 1.624 | 0.107 |
| Residue | 64 | 10112 |  |  |
| Significant at 5% level |  |  |  |  |

**Table I. Two-way ANOVA for color stability.**

| **Effect** | ***df*** | **Mean** | **F** | **Value p** |
| --- | --- | --- | --- | --- |
| **Between groups** |  |  |  |  |
| Groups | 3 | 0.132 | 7.182 | 0.003* |
| Residue | 16 | 0.018 |  |  |
| **Within Groups** |  |  |  |  |
| Time | 3 | 0.047 | 2.883 | 0.045* |
| Groups*Time | 9 | 0.029 | 1.824 | 0.088 |
| Residue | 48 | 0.016 |  |  |
| Significant at 5% level |  |  |  |  |

**Table J. Means and the standard deviation of color stability for all groups evaluated.**

|  | **Groups** | **Mean** | **Standard deviation** |
| --- | --- | --- | --- |
| **7 days** | DW | 0.5253 | 0.08178 |
|  | DS | 0.4820 | 0.13090 |
|  | PS | 0.3238 | 0.09858 |
|  | LS | 0.5038 | 0.15977 |
|  | Total | 0.4587 | 0.13808 |
| **14 days** | DW | 0.6298 | 0.13961 |
|  | DS | 0.2918 | 0.14332 |
|  | PS | 0.4378 | 0.14906 |
|  | LS | 0.5303 | 0.06824 |
|  | Total | 0.4724 | 0.17431 |
| **21 days** | DW | 0.6382 | 0.08903 |
|  | DS | 0.3776 | 0.13400 |
|  | PS | 0.4943 | 0.07689 |
|  | LS | 0.6208 | 0.17993 |
|  | Total | 0.5327 | 0.15874 |
| **28 days** | DW | 0.5751 | 0.15909 |
|  | DS | 0.5415 | 0.18718 |
|  | PS | 0.5501 | 0.09442 |
|  | LS | 0.5732 | 0.08999 |
|  | Total | 0.5599 | 0.12848 |
